# Evaluation of therapeutic radiographer contouring for magnetic resonance image guided online adaptive prostate radiotherapy

**DOI:** 10.1016/j.radonc.2022.109457

**Published:** 2023-03

**Authors:** Gillian Adair Smith, Alex Dunlop, Sophie E. Alexander, Helen Barnes, Francis Casey, Joan Chick, Ranga Gunapala, Trina Herbert, Rebekah Lawes, Sarah A. Mason, Adam Mitchell, Jonathan Mohajer, Julia Murray, Simeon Nill, Priyanka Patel, Angela Pathmanathan, Kobika Sritharan, Nora Sundahl, Alison C. Tree, Rosalyne Westley, Bethany Williams, Helen A. McNair

**Affiliations:** a The Royal Marsden NHS Foundation Trust, London, United Kingdom; b The Institute of Cancer Research/The Royal Marsden NHS Foundation Trust, London, United Kingdom; c Joint Department of Physics at the Royal Marsden and The Institute of Cancer Research, United Kingdom; d Clinical Trials and Statistic Unit, The Institute for Cancer Research, London, United Kingdom

**Keywords:** Prostate radiotherapy, MR-Linac, Radiographer contouring

## Abstract

**Background and Purpose:**

The implementation of MRI-guided online adaptive radiotherapy has facilitated the extension of therapeutic radiographers’ roles to include contouring, thus releasing the clinician from attending daily treatment. Following undergoing a specifically designed training programme, an online interobserver variability study was performed.

**Materials and Methods:**

117 images from six patients treated on a MR Linac were contoured online by either radiographer or clinician and the same images contoured offline by the alternate profession. Dice similarity coefficient (DSC), mean distance to agreement (MDA), Hausdorff distance (HD) and volume metrics were used to analyse contours. Additionally, the online radiographer contours and optimised plans (n = 59) were analysed using the offline clinician defined contours. After clinical implementation of radiographer contouring, target volume comparison and dose analysis was performed on 20 contours from five patients.

**Results:**

Comparison of the radiographers’ and clinicians’ contours resulted in a median (range) DSC of 0.92 (0.86 – 0.99), median (range) MDA of 0.98 mm (0.2–1.7) and median (range) HD of 6.3 mm (2.5–11.5) for all 117 fractions. There was no significant difference in volume size between the two groups. Of the 59 plans created with radiographer online contours and overlaid with clinicians’ offline contours, 39 met mandatory dose constraints and 12 were acceptable because 95 % of the high dose PTV was covered by 95 % dose, or the high dose PTV was within 3 % of online plan. A clinician blindly reviewed the eight remaining fractions and, using trial quality assurance metrics, deemed all to be acceptable.

Following clinical implementation of radiographer contouring, the median (range) DSC of CTV was 0.93 (0.88–1.0), median (range) MDA was 0.8 mm (0.04–1.18) and HD was 5.15 mm (2.09–8.54) respectively. Of the 20 plans created using radiographer online contours overlaid with clinicians’ offline contours, 18 met the dosimetric success criteria, the remaining 2 were deemed acceptable by a clinician.

**Conclusion:**

Radiographer and clinician prostate and seminal vesicle contours on MRI for an online adaptive workflow are comparable and produce clinically acceptable plans. Radiographer contouring for prostate treatment on a MR-linac can be effectively introduced with appropriate training and evaluation. A DSC threshold for target structures could be implemented to streamline future training.

## Highlights

• Radiographer prostate contours are comparable to clinicians.
• Prostate and organs at risk can be contoured online by radiographers.
• Plans created from radiographer contours are clinically acceptable.
• A DSC > 0.90 between gold standard and observer, results in a clinically acceptable plan.

The implementation of magnetic resonance image guided online adaptive radiotherapy (MRIgART), using a MR-linac (MRL), has facilitated tailoring of radiotherapy to daily changes in anatomy due to superior soft tissue definition with MRI and the ability to adapt online [1], [2]. The potential to dose escalate whilst reducing the dose to normal tissues and improving the therapeutic ratio becomes a reality [3]. However, early adopters have reported an increase in staff required to be present at time of treatment delivery [4], [5], which can become a barrier to implementation when combined with increased treatment times of approximately 45 minutes [6]. The need to recontour the target and organs at risk (OAR) online requires the clinician to be present at the time of treatment. The optimisation of MRIgART offers an opportunity to re-define staff roles and responsibilities. The role of the therapeutic radiographer, required to deliver radiotherapy, has previously been successfully extended for tasks originally performed by a clinician. For example, approving port films [7], image verification for stereotactic radiotherapy [8], and plan selection from a library of plans [9], [10]. MRIgART presents the opportunity to extend the role further and relieve the clinician from contouring.

Consistency between observers contouring the prostate has been studied in both CT and MRI. The most common variable used in comparison is volume [11]. It has been well established that the use of MRI instead of, or in combination with, CT reduces interobserver variability [12], [13]. Education programmes have also been shown to improve consistency; prostate volume contouring agreement increased by 16 % with the use of anatomical atlases and practical sessions [14]. All of these studies were offline using clinicians as the observers. Studies comparing radiographers and clinicians using MRI are few but illustrate equivalence [15]. To the authors’ knowledge, the clinical significance, by comparing and measuring dose as a parameter has been rarely used.

During MRIgART, pressure is often experienced by the professional performing their section of the pathway because of both being observed by other members of the team and being aware of the patient remaining still on the bed for an extended period of time [16]. The first study reporting evaluation of online prostate contours on MRI assessed 150 structures contoured independently online by eight radiation technologists (RTTs) [17]. The contours drawn by the RTTs were judged subjectively offline by the clinical oncologists and amendments made where necessary. The contours were deemed acceptable, and therefore unchanged, in 94.2 % of fractions. and dice similarity coefficient (DSC) scores of ≥ 0.98 were achieved. A direct comparison of independent radiographer and clinicians’ contours would be more representative of clinical practice. Dose analysis was only performed for contours deemed as outliers (n = 7) with one fraction calculated as underdosed. OAR doses were not assessed but can have significant impact and may compromise planning target volume (PTV) coverage where OARs are adjacent to the PTV.

We implemented a training programme for radiographers and have directly compared independent radiographers’ and clinicians’ online defined contours with offline contours on the same image. A dosimetric evaluation was also performed using the online-optimised plan created from online radiographer defined targets and OARS, with the offline clinician defined targets and OARs.

## Materials and Methods

### Training programme

Therapeutic radiographers underwent a formal departmental contouring training programme which included offline contouring, online observations and supervised online contouring of target and OAR structures on T2 weighted MRI (Appendix A).

### Pre-treatment

Patients referred for radiotherapy to the prostate, 60 Gy in 20 fractions, treated as per PERMIT trial (NCT03658525) or PACE trial (NCT01584258), on the MRL were included in this service evaluation, approved by local committee for clinical research. All patients consented to have their images used for research. Naming conventions differed between trials (Appendix B), therefore for the purposes of this paper, the prostate plus 1 cm proximal SV target is referred to as high dose CTV and the prostate plus 2 cm proximal SV is referred to as the low dose CTV. The PTVs were created with the respective CTV plus isotropic margins of 0.3 cm for high dose PTV (6000 cGy) and 0.6 cm for low dose PTV (4700 cGy).

A planning CT (Siemens Confidence, Erlangen, Germany), with 1.5 mm slice thickness, and T2-weighted (T2w) planning MRI (Siemens Magnetom Aera 1.5 T, Erlangen, Germany) were acquired for each patient. Enemas were used for two days prior to planning scans, plus 1.5 hours prior to CT. Patients were asked to drink 350 ml of water 45 minutes before each planning scan. Prostate, SV and OARs were delineated on the CT by a clinician, aided by the fused planning MRI. A reference plan for the first fraction was generated (Monaco TPS, Elekta AB, Stockholm, Sweden, V5.40.01) on the planning CT using a 7-field intensity-modulated radiotherapy (IMRT) simultaneous integrated boost technique to treat the two PTVs to different dose levels [18]. A new reference plan for fraction two onwards was created using the MRI acquired on fraction one, and associated structures. This enabled MR to MR registration post fraction one, improving the speed of registration and accuracy of structure propagation.

### Online workflow

Patients were instructed to use an enema 1.5 hours prior to the first 10 fractions, and drink 350 ml water 30 minutes prior to every fraction. After patient set-up [19] a T2w MRI was acquired (MRI^session^) and registered to the reference image (planning CT for fraction one and MRI^session^ for fraction two onwards). The structures from the reference image were deformably propagated onto the MRI^session^, except the bladder which was rigidly propagated in anticipation of intra-fraction filling. The prostate, SV, and OARs within 2 cm of the high dose PTV were amended as necessary on the MRI^session^ and a daily online plan optimised using reference plan parameters (Appendix B). A second T2w MRI (MRI^verification^) was acquired and overlaid with the MRI^session^ to check for any significant change in anatomy during the planning stage and a positional correction was performed if necessary [18].

### Contouring

Fraction one was contoured by a clinician, thereafter trained radiographers contoured online for one week, followed by clinicians contouring the next week, repeating with each profession contouring online for 10 fractions each. A patient specific contouring guide (Appendix C) was created and visible at the treatment terminal daily to inform target delineation.

### Target volume comparison

Each MRI^session^, associated structure set, and online optimised plan were copied offline and structures that had been amended online were reset to the original propagation to recreate the online situation. Clinicians or radiographers were instructed to contour the MRI scans offline with the speed and accuracy of the online environment, although offline speed was not measured. This resulted in all fractions being contoured twice: once online by one profession, and once offline by the other profession. Scans were assigned to ensure a variation of radiographers and clinicians were compared. Interobserver variability was assessed using DSC, MDA, HD and volume metrics (ADMIRE, Research 2.0 Elekta, Stockholm, Sweden).

### Dose analysis

The online radiographer optimised plan was overlaid with the offline clinicians’ contours and dose statistics extracted for the clinician contoured target and OARs.

Based on previous work [20] we expect the mean D98% for the high dose PTV to be > 5730 cGy when plans optimised using radiographer contours are recalculated on the clinician defined contours. To show this, assuming the standard deviation of dose to be 1.56 and dose under the alternative is 5580 cGy (mandatory goal), 57 images would be required to achieve 80 % power (using one-sample t-test, alpha 5 %, one-sided). We assumed images from the same patient are independent. As 10 images would be analysed per patient, images from six patients were required.

Dosimetric success was defined when at least one of the following criteria was achieved:

(i) Offline target (contoured by clinicians) meets all mandatory dose constraints when overlaid with radiographer’s online derived plan
(ii) 95 % of the offline high dose PTV received 95 % of the prescription, as per ICRU guidelines [21]
(iii) In cases where the target coverage was deliberately compromised to achieve OAR dose constraints then offline target dose was within 3 % of online target dose.

If none of the above criteria was achieved, further evaluation of the relevant contours was performed by a consultant clinical oncologist. The MRIs and associated structure sets were anonymised and saved together with images and structure sets which achieved criteria (i), (ii) or (iii). The consultant clinical oncologist blindly reviewed all the prostate and SV contours using national PACE trial (NCT01584258) quality assurance (QA) criteria to determine if contouring had deviated from the “gold standard” and if deviations were likely to either increase side effects or decrease tumour control.

For the OAR analysis, as there were minor differences in OAR dose constraints used between trials (Appendix B), we used a concept of missed versus achieved OAR constraints.

### Dose vs DSC

A benchmark for future radiographer contouring was explored. Previous data [20] indicated radiographer contoured target structures (high dose CTV) with a minimum DSC of 0.9 when compared to a clinician contour, produced plans which met mandatory dose constraints and would therefore be clinically acceptable. The DSC for each online radiographer contoured, and offline clinician contoured, fraction was compared with the offline dose to the high dose PTV, with focus on the mandatory PTV constraint of D98 > 5580 cGy.

### Contouring time

The online contouring time of each profession was recorded from start of target and OAR editing to end of editing.

### Evaluation after clinical implementation

Subsequent to clinical implementation of online radiographer contouring, the volumetric and dosimetric analysis described above was undertaken for one fraction per week of the next five patients to be treated on the MRL*.*

## Results

Seven patients were initially identified. One patient was excluded because a clinician was present for every fraction due to difficulties visualising boundaries between bowel, SV and rectum on T2w MRL-acquired MRI. Data from six patients were analysed.

A total of 117 fractions were delivered on the MRL, with one patient receiving three non-adaptive fractions on a CBCT-linac due to machine breakdown. Online contouring was performed by a group of four radiographers (59 fractions) and seven clinicians (58 fractions). The median DSC between the clinician and radiographer high dose CTV was ≥ 0.9 and the median MDA was < 1 mm for all 117 fractions. The median HD was variable between patients (Table 1).

**Table 1:** Volume comparison (median(range)) of 117 high dose CTV structures, prostate plus 1 cm proximal SV.

Patient	DSC	MDA (mm)	HD (mm)	Radiographer contoured volume (cm^3^)	Clinician contoured volume (cm^3^)
1	0.93 (0.88 – 0.95)	0.9 (0.7 – 1.7)	7.4 (3.6 – 11.5)	69.0 (62.7 – 72.3)	65.7 (58.5–72.4)
2	0.91 (0.89 – 0.99)	1.2 (0.2 – 1.6)	6.4 (2.5 – 9.1)	65.6 (62.5 – 70.9)	65.0 (60.3–70.8)
3	0.90 (0.86 – 0.94)	1.0 (0.7 – 1.6)	5.3 (3.9 – 10.9)	32.4 (28.8 – 36.6)	32.8 (28.9–40.8)
4	0.90 (0.88 – 0.93)	1.0 (0.7 – 1.3)	6.4 (4.0 – 9.0)	39.4 (34.4–43.6)	39.0 (33.8 – 42.4)
5	0.92 (0.89 – 0.95)	0.8 (0.5 – 1.2)	5.1 (3.3 – 7.3)	29.9 (26.9 – 32.0)	30.2 (25.6 – 32.4)
6	0.94 (0.92 – 0.95)	1.0 (0.8 – 1.3)	6.7 (4.7 – 9.6)	103.2 (92.5 – 108.2)	104.1 (91.5 – 110.4)
Overall	0.92 (0.86–0.99)	0.98 (0.2–1.7)	6.3 (2.5–11.5)		

There was no significant difference in volume size between the two groups (p = 0.47). A slight trend towards larger contours during treatment was seen with a median (range) relative volume difference between first and subsequent fractions of radiographers’ and clinicians’ contours of 4.2 % (-3.5 % to 17.7 %) and −0.13 % (-16 % to 22.1 %) respectively.

Of the 59 plans created with radiographer online contours, 51 met the pre-defined dosimetric success criteria. 39 met the mandatory dose constraints (success criteria i) and a further three were acceptable because 95 % of the high dose PTV was covered by 95 % dose (success criteria ii). The target coverage was deliberately compromised to achieve mandatory bowel dose constraints in 9 of the 59 plans, all from patient 6. However, as the offline high dose PTV (clinician contoured) was within 3 % of online dose for all 9 fractions it was deemed acceptable (success criteria iii).

**Fig. 1:**
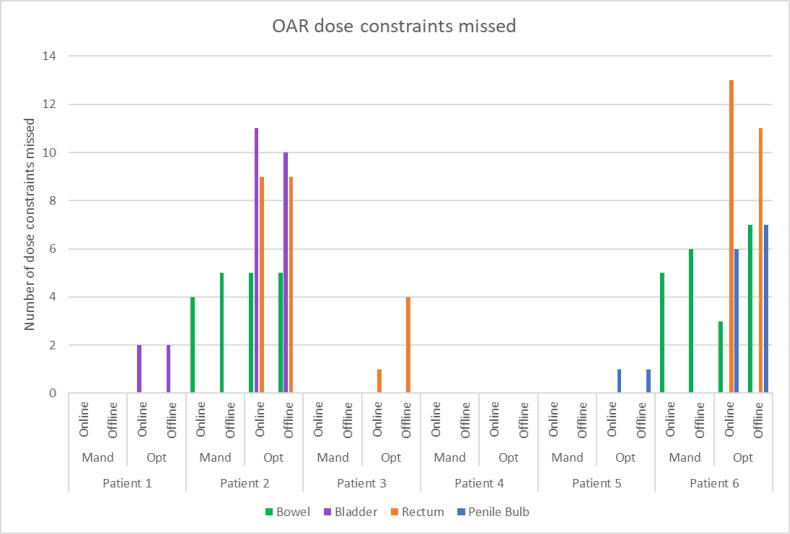
Number of organs at risk dose constraints missed when radiographers contoured online compared to clinicians contouring offline.

The remaining eight MRIs and associated structure sets were deemed to be clinically acceptable following review. Specific feedback for each set of contours included advice to further improve the contour in three cases (one of the failed plan cohort and two of the acceptable planned cohort). Evaluation of missed OAR dose constraints was completed for the 59 radiographer online contoured fractions. For Patients 1, 3, 4 and 5 all mandatory dose constraints were achieved and a maximum of 4 optimal dose constraints were missed (Fig. 1).

**Fig. 2:**
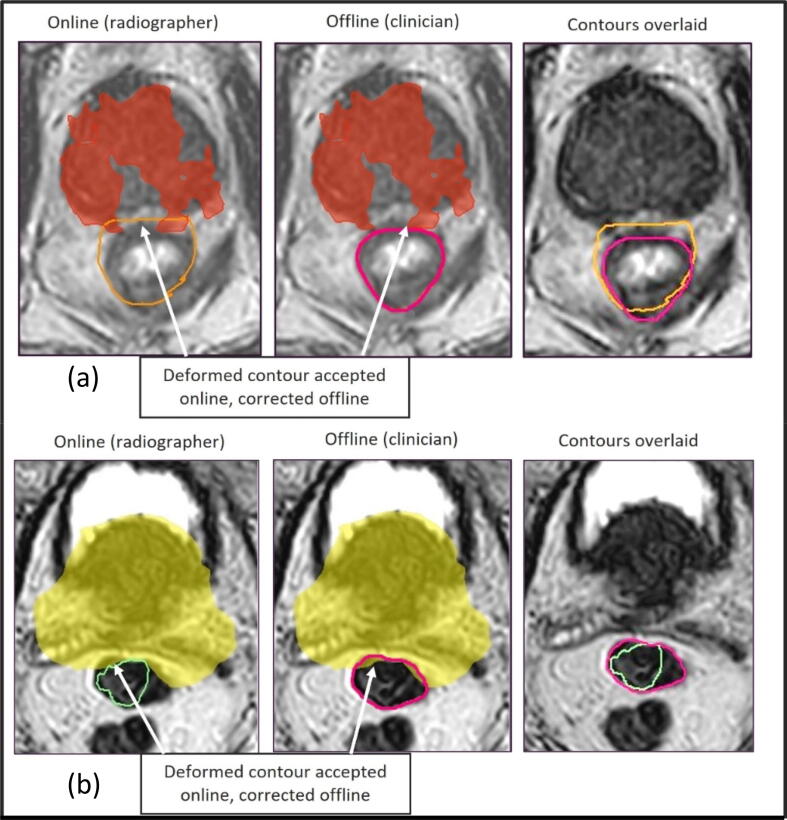
(a) Online (orange) and offline (pink) rectum contours. 6000 cGy isodose shown in red (b) Online (green) and offline (pink) bowel contours. 5200 Gy isodose shown in yellow.

Mandatory bowel dose constraints were intentionally exceeded for online fractions for patient 6 and therefore exceeded offline. Two fewer optimal rectum dose constraints were missed offline because the deformed contour was accepted online but corrected offline (Fig. 2a), resulting in a better estimate of dose delivered. It is therefore likely that this dose constraint was met both online and offline.

For patient 2, after fraction 13, bowel dose analysis was performed. It was determined that, even if the same part of the bowel were to receive the prescription dose every fraction for the remaining fractions, the mandatory bowel tolerance dose constraint would be achieved over the entire course of treatment. Therefore, from fraction 14 onwards, the dose to the bowel structure was no longer optimised online, resulting in failing bowel mandatory dose constraints. We can also see that, for one fraction, one additional mandatory bowel dose constraint was missed offline due to the deformed contour being accepted online but corrected offline (Fig. 2b). However, since the bowel dose analysis resulted in exclusion of bowel dose constraints from online optimisation the deformed contour would have been accepted, particularly due to time pressure in the online environment.

**Fig. 3:**
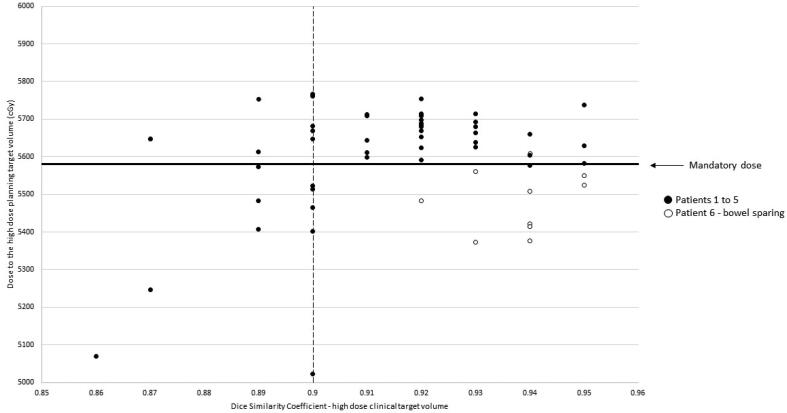
Comparison of high dose CTV DSC to high dose PTV dose, in plans where the online, radiographer contoured plan, is overlaid with the offline clinician structures.

A target DSC (high dose CTV) greater than 0.9 produced plans which met mandatory dose constraints to the high dose PTV (Fig. 3), excluding the patient for whom target coverage was deliberately compromised to meet mandatory bowel dose constraints (white data points). DSC between 0.85 and 0.9 produced plans meeting (n = 10), and not meeting (n = 10), mandatory dose constraints.

Radiographers had a shorter median contouring time for four patients. Clinicians had a shorter median contouring time for patient 6, who required extensive bowel recontouring daily, and patient 3, who had a transurethral resection of the prostate (TURP) (Table 2).

**Table 2:** Median (range) online contouring time for clinicians and radiographers.

Patient	Clinicians online mm:ss	Radiographers online mm:ss
1	**11:00** (08:27 – 18:30)	**09:52** (07:59 – 18:21)
2	**11:44** (08:56 – 19:03)	**10:50** (08:21 – 15:48)
3	**09:31** (06:22 – 11:29)	**10:18** (05:44 – 19:28)
4	**08:42** (06:04 – 11:57)	**08:15** (06:06 – 13:38)
5	**09:36** (07:20 – 13:27)	**08:49** (06:01 – 16:49)
6	**15:52** (11:15 – 26:21)	**16:27** (13:05 – 25:29)

The results for the 20 fractions (5 patients, 1 fraction per week) analysed after clinical implementation were consistent with previous findings. The median (range) DSC between clinician and radiographer high dose CTV was 0.93 (0.88–1.0). The median (range) MDA was 0.8 mm (0.04–1.18), and median (range) HD was 5.15 mm (2.09–8.54). There was no significant difference in volume size between the two groups (p = 0.550).

Of the 20 plans created using radiographer online contours overlaid with clinicians’ offline contours, dosimetric success criteria (i), (ii) or (iii) was achieved in 18 cases. The remaining two high dose CTVs created online by radiographers were smaller than the clinicians’ offline contours (DSC 0.94 and 0.88, volume difference 3.2 cm^3^ and 5.9 cm^3^). These contours were deemed acceptable following review by a clinician without knowledge of dose analysis. All OAR mandatory dose constraints were achieved, a total of nine optimal dose constraints (over 3 patients) were missed.

## Discussion

Therapeutic radiographers successfully contoured target and OARs for prostate MRIgART resulting in clinically acceptable plans in terms of dose delivered to the target and OARs. This is the first study, to our knowledge, comparing the dosimetric impact of contouring variations in an online situation between clinicians and radiographers. Our direct comparison of independent radiographer and clinicians’ contours adds strong evidence to existing studies that contouring the prostate in an online setting can be performed by radiographers [17]. We have shown this both in an evaluation where radiographers contoured 50 % of fractions online (117 contours and 59 plans from 6 patients) and the subsequent evaluation of clinical implementation (20 contours and plans from 5 patients). The effect of contouring variation on OARs and toxicity has been previously investigated between five clinicians and five patients [22]. Although statistically significant differences were found between the prostate contours, mainly at the apex and seminal vesicles, there was no statistically significant differences found between the estimates of bladder (p = 0.1) and rectal (p = 0.09) normal tissue complication probability [23]. We found differences in OAR constraints missed, mainly due to the online practice of only recontouring OARs within 2 cm of the PTV where the propagated contour was deemed inadequate for online adaptive planning [5] (Fig. 2). In the offline environment OARs were recontoured more accurately within 2 cm of the PTV, suggesting that radiographers and clinicians did not entirely abide to the speed and accuracy of the online environment instructions.

The variation in contours of the eight fractions that did not meet the pre-defined criteria for dosimetric success, was mainly seen at the apex and base. This is consistent with many studies which have shown the apex is prone to greater interobserver variability [12]. Although MRI provides superior soft tissue definition, these areas remain less well defined [15], [17]. However, the limitations of this study need to be considered. For example, a larger offline contour than online would immediately result in reduced dose to the offline clinician contoured prostate. Here the offline contour volume in seven of the eight cases where the pre-defined criteria was not met, was greater than the online contour. On examination, by a blinded expert, the eight contours were deemed clinically acceptable and in fact resulted in less feedback than the contours which had met the dose constraints. This highlights the fact that there is no ‘ground truth’ when investigating contouring variability. To create robust comparisons, we have used MDA and HD to provide an indication of shape and boundary agreement, which DSC alone does not provide. Comparisons with other studies are made difficult because of variation in metrics used, for example volume-only comparisons [23], [24] and contouring variation [25], [26]. Where published, our median MDA of < 1 mm is similar [27], [15]. The trend of increasing volume may be due to prostate swelling during treatment [28].

Comparing one radiographer directly with one clinician means any interobserver variability in the groups would have an impact on results. The range in volume was greater amongst the clinicians and the volume of the radiographers’ contours were within the clinicians’ volume range in three of the six patients. Although there were more clinicians contouring, which could have contributed to the greater interobserver variability, there may be other influencing factors such as training and information available whilst contouring. Although a contouring guide with specific instructions to guide delineation (Appendix C) was visible online for all fractions it may be that the clinicians are more independent in their thinking and not all had experience of the training programme; three were instrumental in the training programme. Individual feedback is an important component of training programmes [10], [29] and the offline simulated and the online observed sessions provided an environment which facilitated individual feedback from one of the three clinicians involved in training which may have improved consistency.

We recommend a training programme and patient-specific instructions to achieve clinically acceptable and consistent online multi-observer contouring. Daily online contouring has been identified as a potential risk for MRIgART but can be minimised by adequate training [30]. For a training programme to be generalisable, it must be remembered that the dosimetric analysis will be dependent on the planning solution and PTV margins used. If implementing a similar procedure in another department, we recommend some form of audit and/or assessment. Competency is difficult to measure, and we investigated a threshold “pass mark” for acceptable contouring. When comparing plans generated using gold standard contours on CT images versus auto segmented contours the mean ± DSC was 0.87 ± 0.03 but the D98 ± 2 % and V95% for prostate and its 3 mm expansion were within 2 % (3 Gy) of each other except for one case with a DSC of 0.82 [31]. The effect of contouring variability on dose volume histogram (DVH) metrics when treating the prostate to 75 Gy was investigated using one CT image and 25 observers. The mean (SD) DSC was 0.838 ± 0.067 with a SD of dose variance of 3.44 Gy leading to the conclusion that the dosimetric impact of contouring variability could not be predicted solely with DSC [32]. However, our study supports the concept [20] that a target structure with a higher threshold DSC of > 0.9, achievable on MR images, produces clinically acceptable plans i.e., which meet mandatory dose goals and constraints. For future training of radiographers, or indeed any observer, a DSC of > 0.9 for 100 % of contours could be deemed a pass for prostate MRIgART. However, since the eight plans that failed dosimetric analysis had clinically acceptable contours when reviewed, a DSC of > 0.9 for 87 % of contours may be acceptable.

Contouring time was similar between radiographers and clinicians and was comparable to published RTT contouring (12.6 +/- 3.8 mins) [17]. Radiographers had a shorter median contouring time for four of the six patients, possibly because of familiarity and confidence with the software. Dedicated time on the relevant treatment planning system may be important for familiarisation of contouring tools. For the two patients where the clinicians were quicker, additional contouring was required for bowel loops abutting the seminal vesicles (patient 6), and a TURP (patient 2). Clinicians may have an advantage with superior anatomy and contouring knowledge and experience, enabling faster decision making in the more complicated cases. Indeed, some cases may always require clinician input, such as the patient excluded, because of difficulty in visualising, or unusual, anatomy. Although, in the future, artificial intelligence may provide a faster solution [33], the contours will require validation, therefore any training implemented will remain applicable. Radiographers’ contouring is a major change in responsibility which requires audit and oversight to maintain standards. However, the clinician remains responsible for contouring the reference plan and recent IR(ME)R 2017 regulations include such a situation as the MRL where radiographers may be responsible for online contouring [34]. In line with the recommendation that *“All radiotherapy departments should have processes that enable optimal target volume delineation and subsequent peer review*” [35], an audit process has been instigated where four sets of contours per patient will be reviewed by a clinician. Whilst contouring requirements for hypo-fractionated cases will be similar to 20 fractions, more consideration regarding plan acceptability thresholds will be required.

## Conclusions

Radiographer prostate and seminal vesicle contours on MRI for an online adaptive workflow are comparable to clinicians’ and produce clinically acceptable online plans. Independent radiographer contouring for prostate treatment on a MR-linac can be effectively introduced with appropriate training and evaluation. A DSC threshold of target structures could be implemented to streamline future radiographers training. Contouring times between the two professions are comparable. Target volume contours will be subject to ongoing audit.

## Declaration of Competing Interest

The authors declare the following financial interests/personal relationships which may be considered as potential competing interests: Dr Helen A McNair reports financial support was provided by National Institute for Health Research and Health Education England. Alison Tree, Angela Pathmanathan, Rosalyne Westley reports a relationship with Elekta Ltd. Alison Tree reports a relationship with Accuray Inc. Alison Tree reports a relationship with Varian Medical Systems Inc. Alison Tree, Sophie Alexander reports a relationship with Cancer Research UK that. Research at The Institute of Cancer Research is also supported by Cancer Research UK under Programme C33589/A28284 and C7224/A28724..

## Figures and Tables

**Table:** Dose metrics for 60 Gy in 20 fractions prostate radiotherapy on the MR-linac.

Trial	Structure	Dose metric						Online Monaco tolerance		

PERMIT trial	PTVpsv_6000	D	98	cGy	>	5700	cGy	-	120	cGy
	(High dose PTV)	D	50		>	5940	cGy			
		D	50		<	6060	cGy			
		D	2	cGy	<	6300	cGy	+	120	cGy
	PTVsv_4700-PTVpsv_6000	D	96		>	4470	cGy			
	(Low dose PTV)	D	50		>	4700	cGy			
	Bladder	V	6000	%	<	5	%	+	10	%
		V	4800		<	25	%			
		V	4000		<	50	%			
	Bowel	V	5200		<	0	cm^3^			
		V	4800	cm^3^	<	0.5	cm^3^	+	5.5	cm^3^
		V	4000	cm^3^	<	17	cm^3^	+	53	cm^3^
	Rectum	V	6000	%	<	1	%	+	2	%
		V	5600		<	15	%			
		V	5200		<	30	%			
		V	4800	%	<	27	%	+	8	%
		V	4000	%	<	38	%	+	12	%
		V	3200	%	<	51	%	+	14	%
		V	2400	%	<	70	%	+	10	%
	Femoral heads	V	4000	%	<	5	%	+	45	%
	Penile bulb	V	2200	%	<	50	%	+	50	%
		V	4800	%	<	10	%	+	90	%
PACE trial	PTVp_6000	D	98	cGy	>	5700	cGy	-	120	cGy
	(High dose PTV)	D	50		>	5940	cGy			
		D	50		<	6060	cGy			
		D	5		<	6300	cGy			
		D	0.1		<	6420	cGy			
	PTVpsv_4700	D	98		>	4465	cGy			
	(Low dose PTV)	D	50		>	4700	cGy			
	Rectum	V	2000		<	85	%			
		V	3000		<	57	%			
		V	4000	%	<	38	%	+	22	%
		V	4800		<	50	%			
		V	5000	%	<	22	%	+	8	%
		V	5600		<	15	%			
		V	6000	%	<	1	%	+	2	%
	Bladder	V	4000		<	50	%			
		V	4800	%	<	25	%	+	25	%
		V	5680	%	<	5	%	+	30	%
		V	6000	%	<	3	%	+	22	%
	Femoral heads	V	4050	%	<	5	%	+	45	%
	Bowel	V	3600	cm^3^	<	78	cm^3^	+	80	cm^3^
		V	4000	cm^3^	<	40	cm^3^	+	70	cm^3^
		V	4400	cm^3^	<	14	cm^3^	+	14	cm^3^
		V	4800	cm^3^	<	0.5	cm^3^	+	5.5	cm^3^
		V	5200		<	0.01	cm^3^			
	Penile bulb	V	2200		<	50	%			

**Figure:**
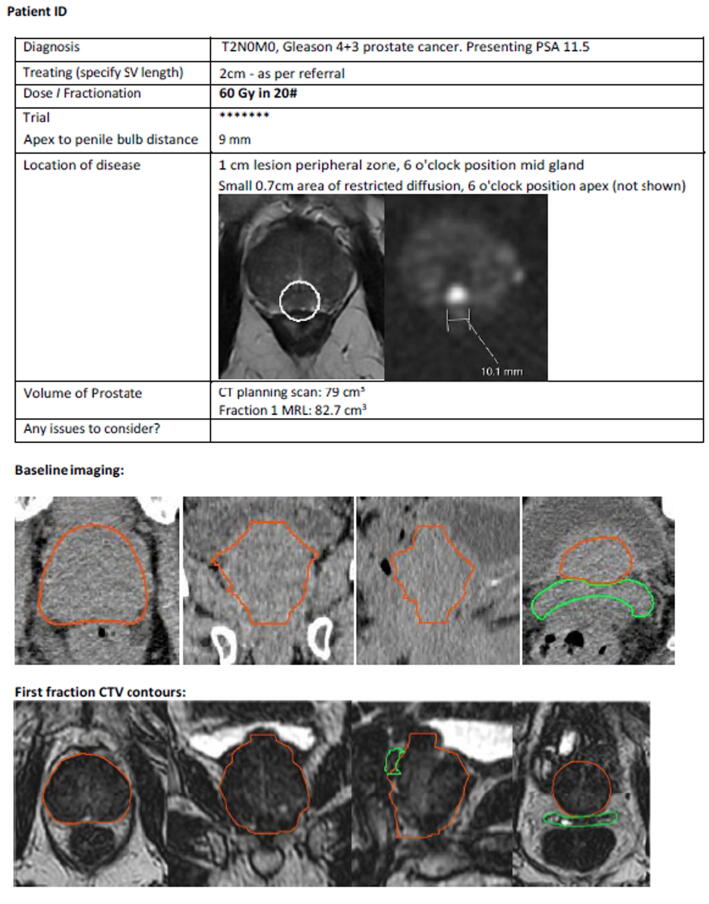
Online contouring guide - Courtesy of K. Sritharan.

## Appendix A

Radiographers were required to undertake the following training before contouring independently online on the MR-linac:

1) Watch presentations, which included revision of the following:
  a. Prostate cancer staging
  b. Prostate anatomy
  c. Contouring considerations (e.g., Appendix C)
  d. Side effects of radiotherapy to the prostate
  e. Effects of bladder filling and rectal changes on decision making
  f. Examples of difficult anatomy, e.g., bowel/sv differentiation
  g. T2w MRI prostate contouring atlas
2) Complete training cases with clinician and individual feedback, at a minimum of:
  a. 5 online observations of clinician led sessions
  b. 5 offline practices reviewed by clinician
  c. 5 online sessions supervised by clinician
